# Oestrogen receptor beta: how should we measure this?

**DOI:** 10.1038/sj.bjc.6600534

**Published:** 2002-09-04

**Authors:** V Speirs, P J Carder, M R J Lansdown

**Affiliations:** Molecular Medicine Unit, Clinical Sciences Building, St James's University Hospital, Leeds LS9 7TF, UK

## Abstract

*British Journal of Cancer* (2002) **87**, 687–687. doi:10.1038/sj.bjc.6600534 www.bjcancer.com

© 2002 Cancer Research UK

## Sir

Since a second oestrogen receptor (ER), ERβ was identified in the middle of the 1990s (Mosselman *et al*, 1996), there has been much effort directed into trying to define its biological role in hormone sensitive tissue such as the mammary gland, particularly at the protein level. In a recent issue of *British Journal of Cancer*, Saunders *et al* (2002) reported immunohistochemical detection of ERβ in 51 breast tumours. In their study cohort, almost all samples were positive for ERβ (48 out of 51; 94%). In an earlier issue of *British Journal of Cancer* (Skliris *et al*, 2001), we too reported immunohistochemical detection of ERβ in a similar sized cohort of 63 breast tumours, and showed much less positive nuclear ERβ immunoreactivity (48 out of 65; 74%). It is interesting to note that both studies used an identical scoring system, based on assessing both staining intensity (scored 0–3) and percentage positivity (scored 0–5), which generates a numerical score ranging from 0–8 (Allred *et al*, 1998). In both instances, a score of >2 was considered positive.

Why such divergent results in such similar sized cohorts? A Scottish–English patient bias seems unlikely (but has not been excluded!). However, it may be pertinent that each study used monoclonal antibodies directed against different parts of ERβ. More recent work from our group has shown a considerable variation in the efficacy of distinct ERβ antibodies to detect receptor protein under different applications (Skliris *et al*, 2002) and Saunders also makes this point in her penultimate paragraph, when referring to other published immunohistochemical studies. It is also perhaps important to remind ourselves that the appropriate threshold for scoring a tumour as ‘positive’ for ERβ will remain unclear until immunohistochemical expression can be correlated with endocrine response in the clinical context. ERβ may well be an important player in the ER signalling cascade but until there is a recognised consensus on the best way to measure it in a clinically meaningful way, the waters will surely remain muddied.

## References

[bib1] AllredDCHarveyMJBerardoMClarkGM1998 Prognostic and predictive factors in breast cancer by immunohistochemical analysis Mod Path 11 155168 9504686

[bib2] MosselmanSPolmanJDijkemaR1996 ER-β: identification and characterisation of a novel human estrogen receptor FEBS Lett 392 4953 8769313 10.1016/0014-5793(96)00782-x

[bib3] SaundersPTKMillarMRWilliamsKMacphersonSBayneCO'sullivanCAndersonTJGroomeNPMillerWR2002 Expression of oestrogen receptor beta (ERβ1) protein in human breast cancer biopsies Br J Cancer 86 250256 11870515 10.1038/sj.bjc.6600035 PMC2375186

[bib4] SklirisGPCarderPJLansdownMRJSpeirsV2001 Immunohistochemical detection of ERβ in breast cancer: towards more detailed receptor profiling? Br J Cancer 84 10951098 11308260 10.1054/bjoc.2001.1721 PMC2363860

[bib5] SklirisGPParkesATLimerJLBurdallSECarderPJSpeirsV2002 Evaluation of seven oestrogen receptor β antibodies for immunohistochemistry, Western blotting and flow cytometry in human breast tissue J Pathol 197 155162 12015738 10.1002/path.1077

